# A resource of data and samples in cognitively unimpaired 60–75‐year‐olds at increased risk of Alzheimer's disease based on their *APOE4* status as heterozygotes or homozygotes

**DOI:** 10.1002/alz70855_107073

**Published:** 2025-12-25

**Authors:** Christopher T. Brown, Jessica Enos, Carolyn Langlois, Trisha L Walsh, Hayley Salata, Yi Su, Vivek Devadas, Jeremy J. Pruzin, Jessica B. Langbaum, Pierre N. Tariot, Eric M. Reiman

**Affiliations:** ^1^ Banner Alzheimer's Institute, Phoenix, AZ, USA; ^2^ University of Arizona College of Medicine Phoenix, Phoenix, AZ, USA; ^3^ ASU‐Mayo Center for Innovative Imaging, Tempe, AZ, USA; ^4^ University of Arizona College of Medicine, Tempe, AZ, USA; ^5^ Banner Alzheimer's Institute and University of Arizona College of Medicine, Phoenix, AZ, USA

## Abstract

**Background:**

The Alzheimer's Prevention Initiative (API)Generation Program studies were sponsored by Novartis and Amgen, in partnership with Banner Alzheimer's Institute and with support from the U.S. National Institute on Aging and Banner. API Generation Study 1 (NCT0256551) was designed to evaluate cognitive, clinical and biomarker effects of the beta‐secretase (BACE1) inhibitor umibecestat and the amyloid beta immunotherapy CAD106 in cognitively unimpaired 60–75‐year‐old *APOE4* homozygotes, including those with or without elevated brain amyloid. API Generation Study 2 (NCT03131453) was designed to evaluate umibecestat's effects in a cognitively unimpaired 60–75‐year‐old *APOE4* carriers (*APOE4* homozygotes and heterozygotes); *APOE4* heterozygotes were required to have elevated brain amyloid.

**Method:**

Participants were recruited from 120 trial sites in24 countries using GeneMatch and other sources. Comprehensive clinical assessments, brain images, and lumbar punctures were performed at screening. RNA, DNA, plasma, serum, and CSF samples were also collected in those who completed screening. The studies were discontinued early due to mild but ultimately reversible cognitive worsening in persons treated with umibecestat.

**Result:**

Together, API Generation Studies 1 and 2 collected and shared screening data from 10,000 volunteers. Data from 2,881 APOE4 homozygote and heterozygote volunteers randomized to the treatment epoch are also available, 52% of whom are amyloid positive based on positron emission tomography (PET) or cerebrospinal fluid (CSF). Aliquots of residual biomaterial including RNA (3,122), DNA (933), Plasma (81,119), Serum (31,977) and CSF (8,104) are also available for sharing. The API Generation Program data and biological samples have received 188 applications and scientific proposals.

**Conclusion:**

The API Generation Program data and biological samples offer researchers a chance to further assess some of the earliest biological and cognitive changes associated with the predisposition to the disease, evaluate initial effects of the two investigational anti‐amyloid treatments, and help inform the design of future prevention trials. Data and samples are available for sharing and can be requested through the Imaging and Data archive at https://ida.loni.usc.edu, the Alzheimer's Disease workbench at https://Alzheimersdata.org/ad‐workbench, or by email at APIData@bannerhealth.com.

